# Supplementary material for: Structural basis of bacterial effector protein azurin targeting tumor suppressor p53 and inhibiting its ubiquitination
Source: Commun Biol. 2023 Jan 17;6:59. doi: 10.1038/s42003-023-04458-1 (PMC9845241; doi:10.1038/s42003-023-04458-1)
Supplement: Supplementary file 2 — Supplementary Information [file 42003_2023_4458_MOESM2_ESM.pdf]

Supplementary Information for

Structural basis of bacterial effector protein azurin targeting tumor suppressor p53 and inhibiting its ubiquitination

Jianjian Hu[1#], Wenxue Jiang[2#], Jiaqi Zuo[1#], Dujuan Shi[2#], Xiaoqi Chen[2], Xiao Yang[2], Wenhui Zhang[1], Lixin Ma[2], Zhu Liu[2], Qiong Xing[2*]

[1] National Key Laboratory of Crop Genetic Improvement, Hubei Hongshan Laboratory, Huazhong Agricultural University, Wuhan 430070, China.

[2] State Key Laboratory of Biocatalysis and Enzyme Engineering, College of Life Sciences, Hubei University, Wuhan 430074, China.

[#] Contributed equally.

* For correspondence:

Qiong Xing (qiongxingnmr@hubu.edu.cn)

This file includes:

Supplementary Figures 1-8

Supplementary Tables 1

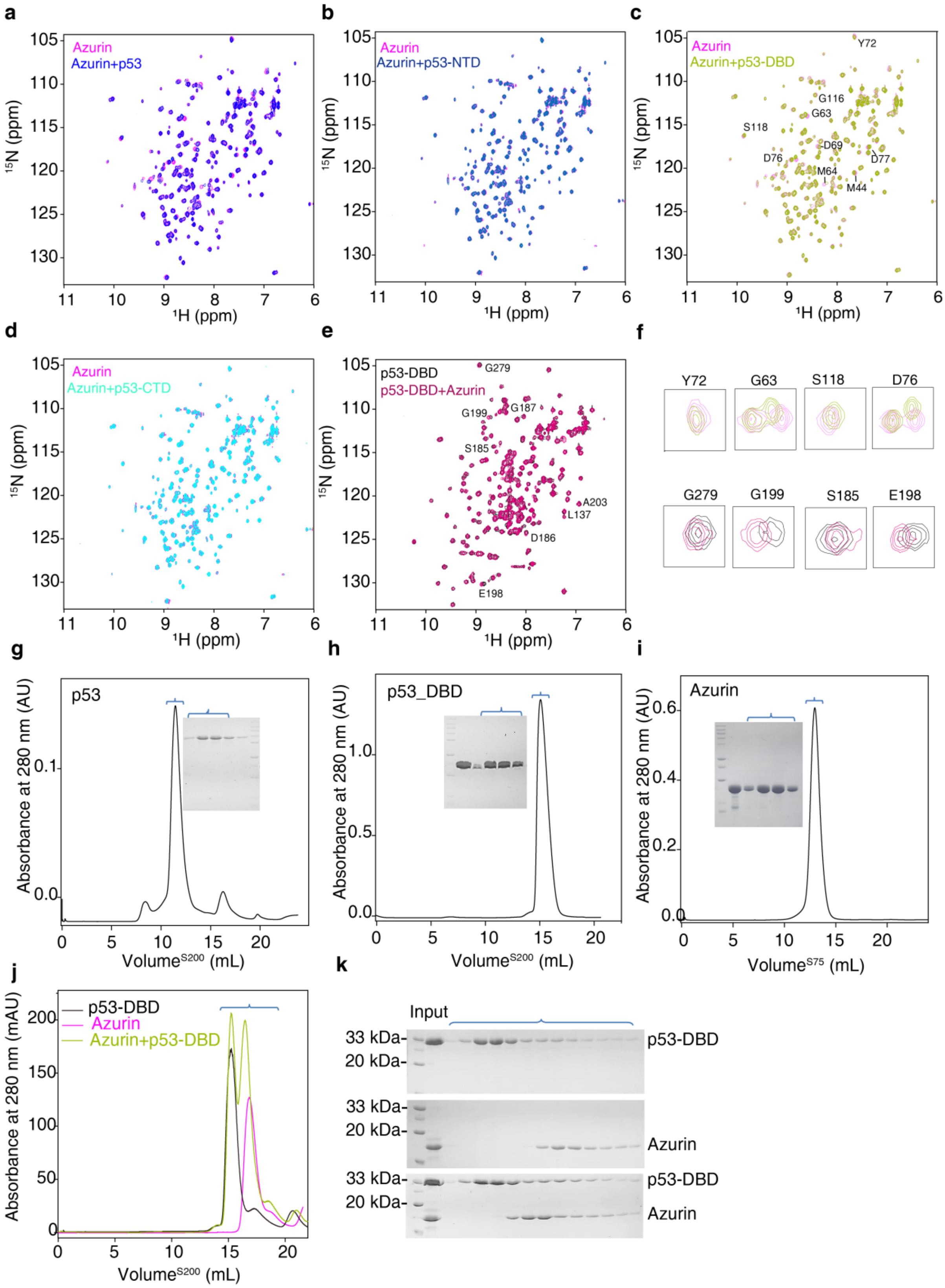

a
Azurin
Azurin+p53
¹⁵N (ppm)
¹H (ppm)
b
Azurin
Azurin+p53-NTD
c
Azurin
Azurin+p53-DBD
Y72
G116
G63
S118
D69
D76
D77
M64
M44
d
Azurin
Azurin+p53-CTD
e
p53-DBD
p53-DBD+Azurin
G279
G187
G199
S185
A203
L137
D186
E198
f
Y72
G63
S118
D76
G279
G199
S185
E198
g
p53
Absorbance at 280 nm (AU)
Volume^S200 (mL)
h
p53_DBD
i
Azurin
Volume^S75 (mL)
j
p53-DBD
Azurin
Azurin+p53-DBD
Absorbance at 280 nm (mAU)
k
Input
33 kDa
20 kDa
p53-DBD
Azurin

Supplementary Figure 1. Interaction between p53 and azurin.

a Overlaid ^{1}H-^{15}N-HSQC spectra of ^{15}N labeled azurin and labeled azurin titrated with unlabeled p53 full-length. The titration of unlabeled p53-NTD, p53-DBD and p53-CTD to ^{15}N labeled azurin is shown in **b, c,** and **d** respectively. **e** The reverse titration of ^{15}N labeled p53-DBD and unlabeled azurin was monitored by NMR in the same way. **f** Highlighted spots of the representative perturbed peaks in the NMR titration spectrum of **c** and **e**. Representative size exclusion chromatography (SEC) profiles as well as SDS-PAGE of azurin, p53, and p53-DBD are shown in **g**, **h** and **i**, respectively. The symbol { in SEC profiles represents the sample loading ranges for the SDS page. **j** Size exclusion analysis of the interaction between azurin and p53-DBD. UV traces for 0.2 mM and 0.5 mL p53-DBD, 0.2 mM and 0.5 mL azurin, and p53-DBD/azurin mixture at 1:1 is colored in black, pink, and dark lime, respectively. **k** SDS pages analysis of the correlated size exclusion chromatography in **j**. The symbol { in SEC profiles reflects the sample loading ranges for SDS page analysis.

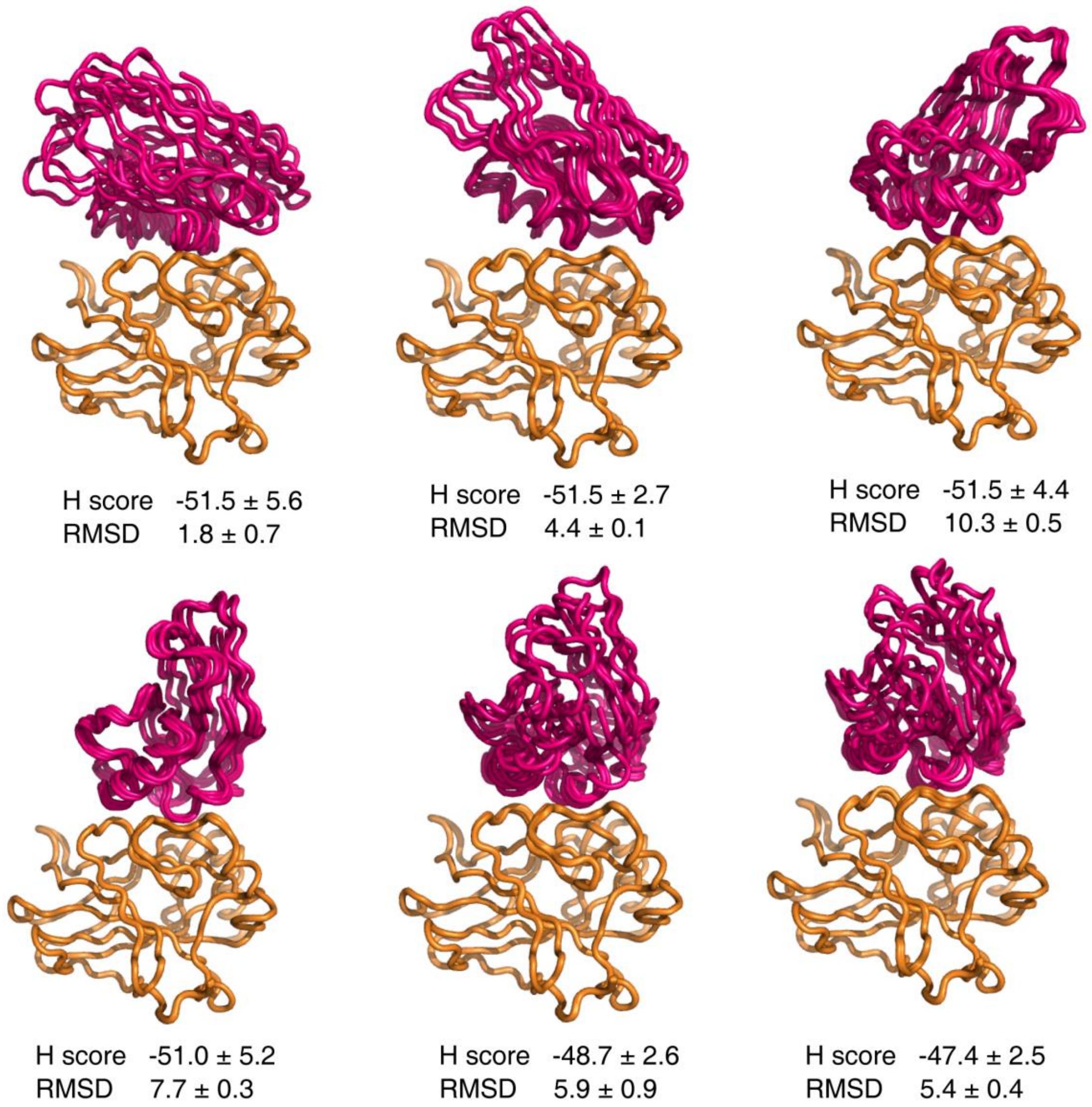

Supplementary Figure 2. HADDOCK model of the azurin-p53 complex using restraints from NMR titration.

The lowest five energy structures from each cluster are presented as cartoon tube with azurin colored in medium violet and p53-DBD colored in orange. The HADDOCK score (H score) and RMSD value of each cluster are shown under the clusters.

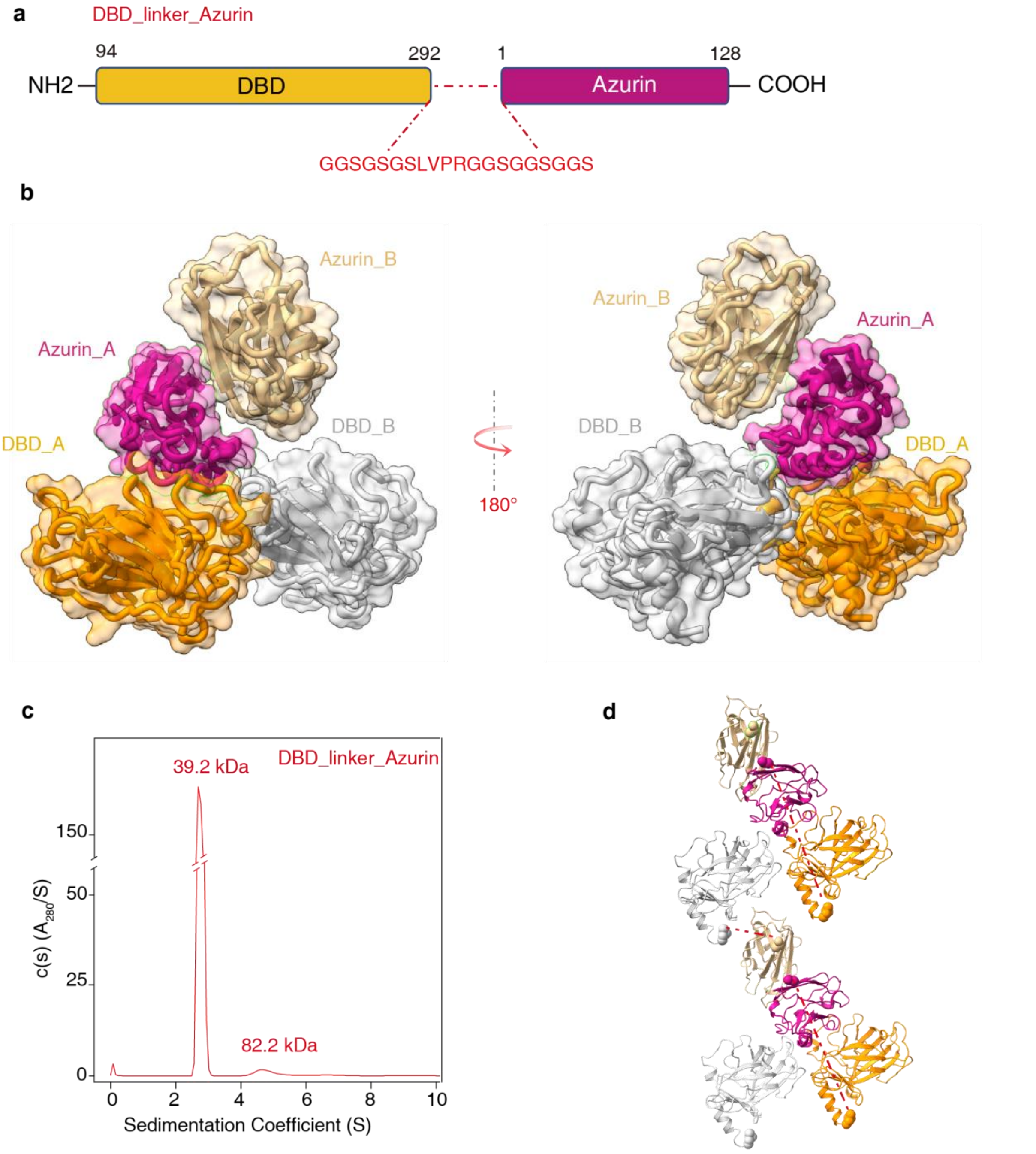

Supplementary Figure 3. Characterization of the crystal structure and interaction of the azurin-p53 complex.

a Representative scheme of the protein fusion constructs used for crystal structure determination. In the construct, azurin is fused to the C-terminal of p53-DBD with a glycine-serine repeated linker. **b** An asymmetric unit of azurin/p53 complex is presented as cartoon and surface. Each unit cell contains two azurin molecules

colored in medium violet and wheat, and two p53 molecules colored in orange and silver. **c** Sedimentation velocity analysis of the p53-DBD_linker_azurin with protein sample of 0.75 mg/mL. Molecular weights in kDa determined from data fitting are included. **d** The theoretical linker region of the DBD-linker-azurin construct in the crystal packing. The linker region is shown as a broken line colored in red.

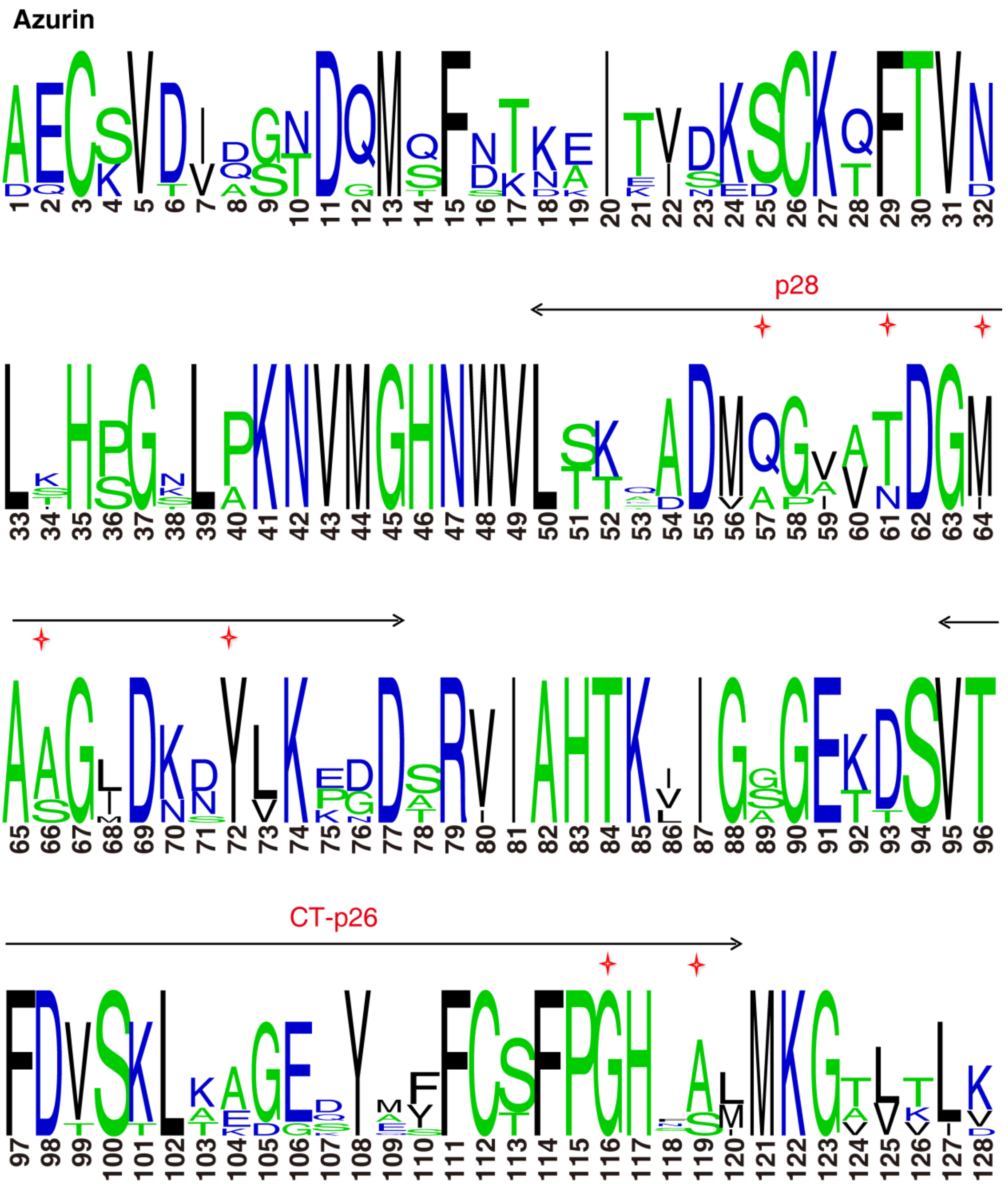

Supplementary Figure 4. Sequence conservation of bacteria azurin

The sequence logo of bacteria azurin was generated with a collection of aligned sequences. The input 20 sequences from different bacteria strains were aligned with Clustal Omega online server (https://www.ebi.ac.uk/Tools/msa/clustalo/). The uniport identifiers of the sequences we used here are sp|P00282, sp|P00285, sp|P00286,

tr|A0A2C9EFK5, sp|P56547, sp|P00279, sp|B3EWN9, tr|A0A3G2NAY1, tr|Q4KJ49, tr|A0A2W0FVA7, tr|A0A2R3IT62, tr|A0A4Y6GKM9, tr|A0A0H2ZI65, tr|A0A5K1S763 tr|A0A2R3IT62, tr|A0A3S4N092, tr|A0A431XAX3, tr|A0A4Y6GMG4, tr|A0A4Y6GMT7, tr|A0A8D9N7S2. Residues involved in the interaction of p53 and azurin were highlighted with a red star above.

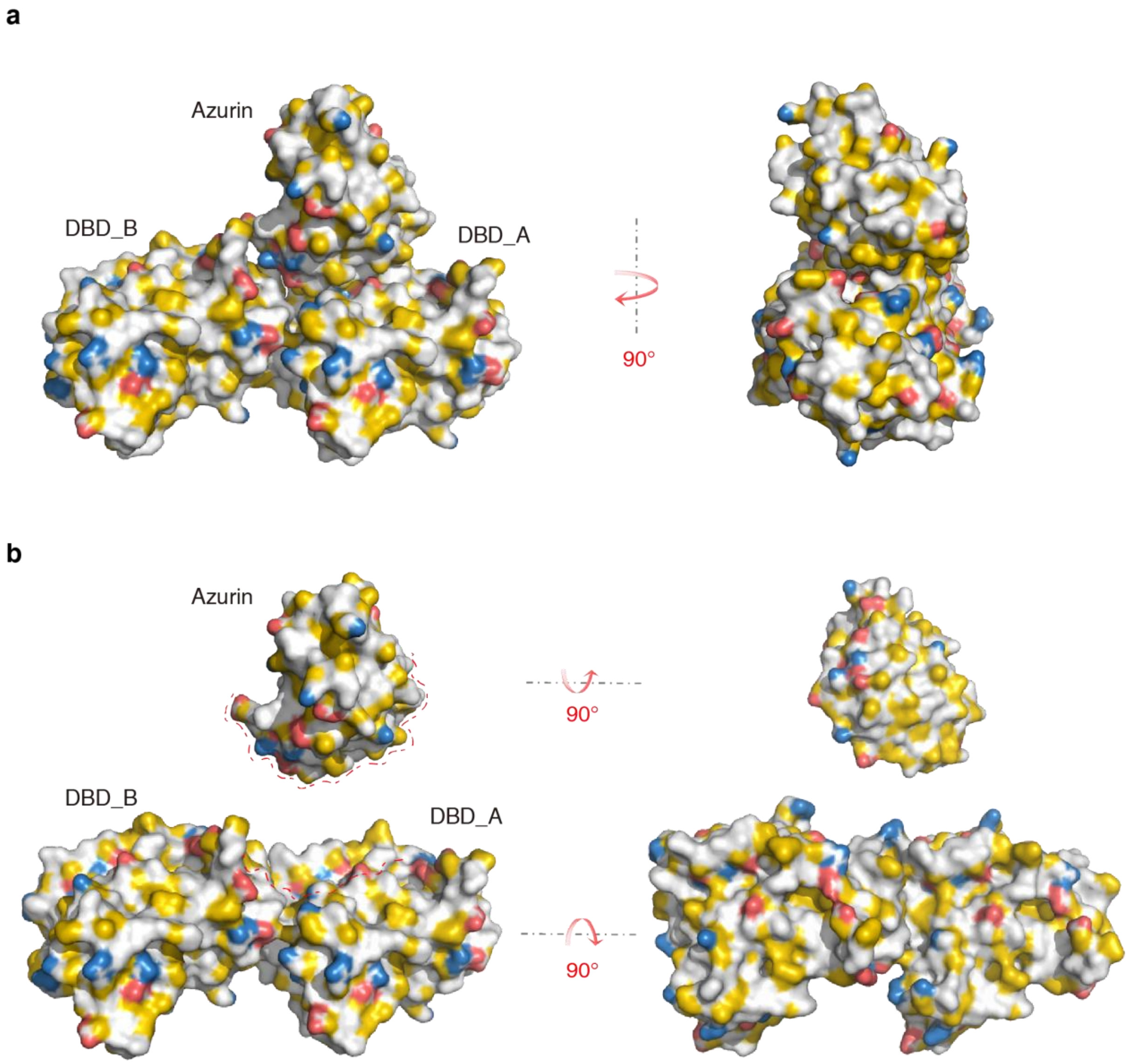

Supplementary Figure 5. Hydrophobicity and hydrophilicity of the azurin, p53-DBD dimer, and the complex.

a Surface rendering of the p53-DBD/azurin complex in the YRB color scheme. In the YRB color scheme, yellow, red, and blue represent hydrophobicity, negative charge, and positive charge, respectively. **b** Surface properties and shape complementarity of p53-DBD dimer and azurin in the context of p53-DBD/azurin complex.

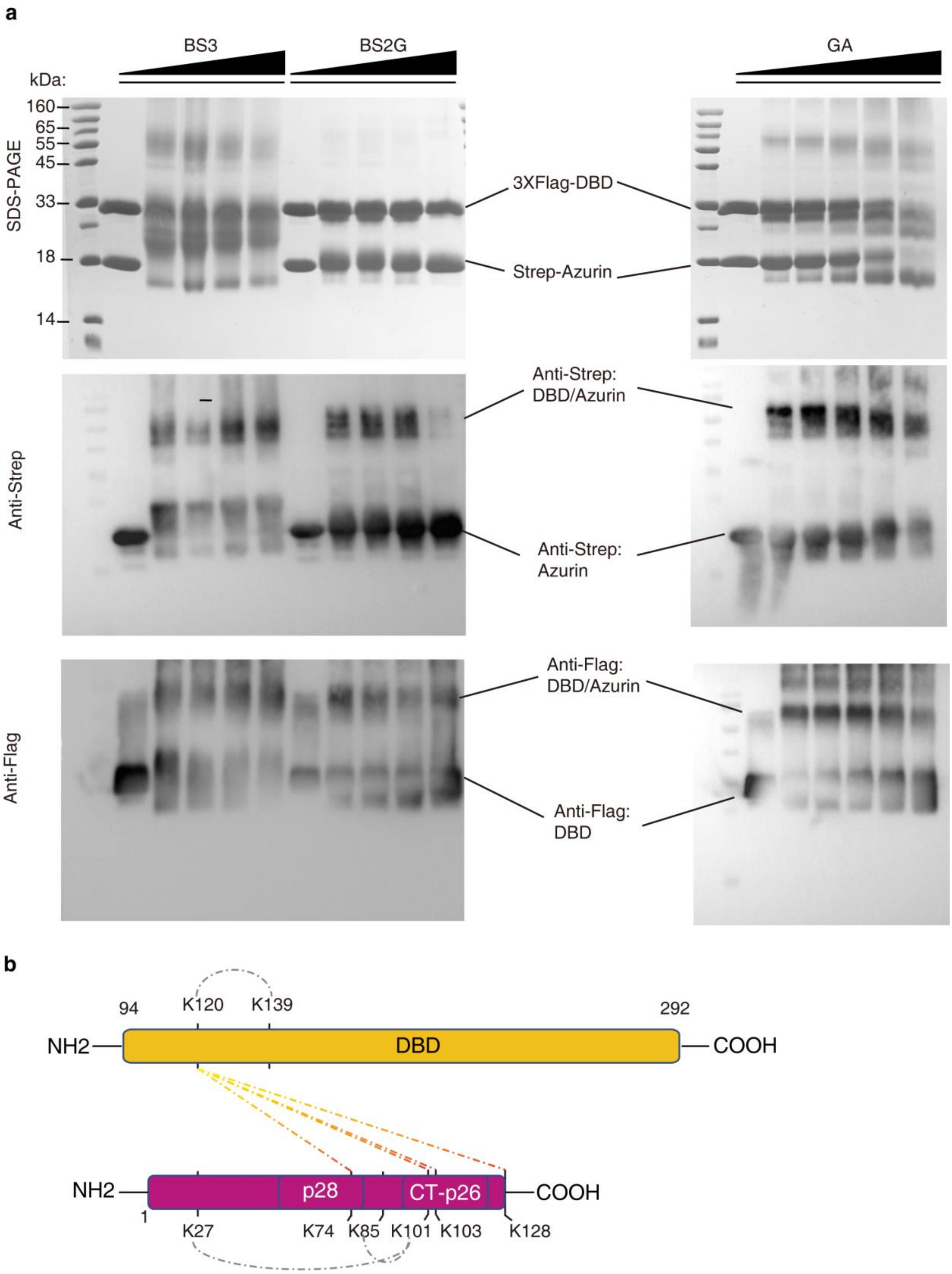

Supplementary Figure 6. Structure validation of the azurin-p53 complex by chemical crosslinking mass spectrometry.

a Crosslinking of the p53-DBD and azurin in solution with three different types of crosslinking reagents, BS3 (bis(sulfosuccinimidyl)suberate), BS2G (Bis [Sulfosuccinimidyl] glutarate) and GA (Glutaraldehyde). **b** Mass spectrometry analysis of the crosslinked pairs in DBD-Azurin complex treated with BS3. Intermolecular crosslinked pairs are plotted with dash line colored in orange, while intramolecular pairs are colored in grey.

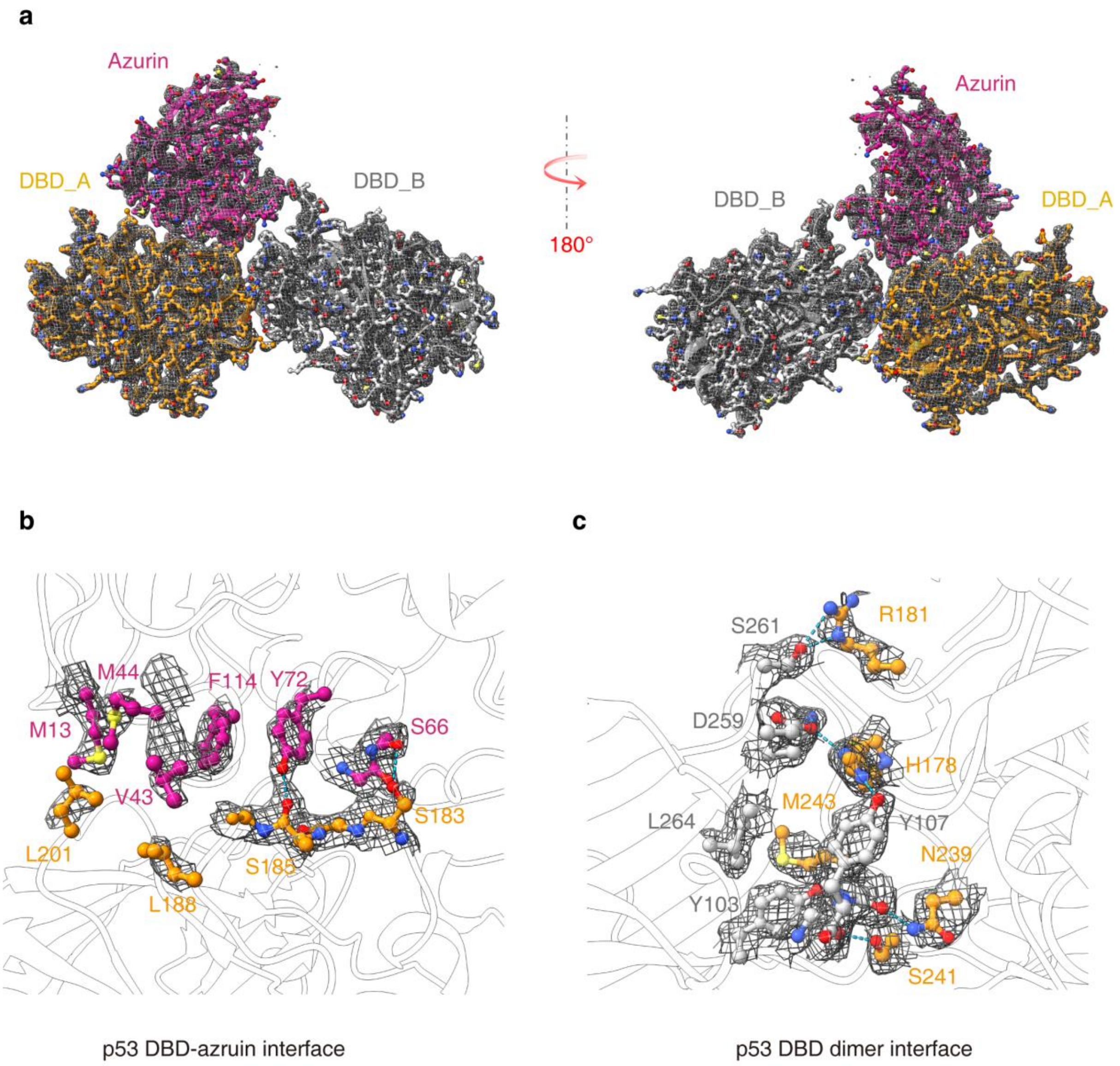

Supplementary Figure 7. Electron density maps of the protein structure.

a Stereo view of azurin in complex with DBD dimer with the 2Fo-Fc electron density map with contour at 1.0 sigma level. **b, c** Stereo view at 1.0 sigma level of the key amino acids in the azurin-p53 interface and p53-DBD dimer interface, respectively.

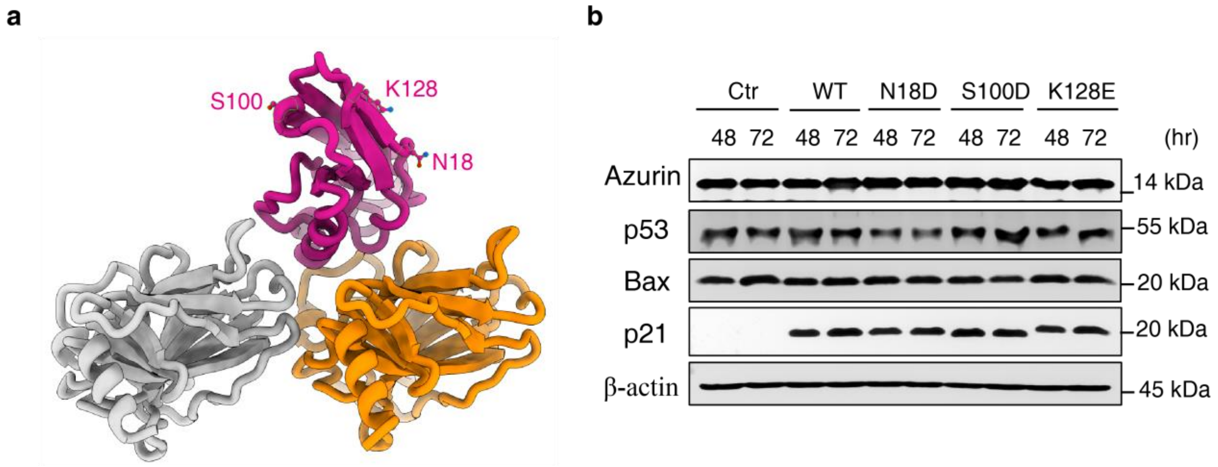

Supplementary Figure 8. Cytoplasmic p53, Bax and p21 levels in cells treated with azurin and its non-effect mutants.

a Amino acids selected on azurin that located far away from the azurin-p53 interface. Three targeted amino acids are shown as sticks and labeled as S100, K128 and N18. **b** Immunoblotting assay shows the effect of azurin and azurin mutants N18D, S100D and K128E on the cytoplasmic level of p53 and its subsequent effectors, Bax and p21. The protein levels of β-actin were shown as control.

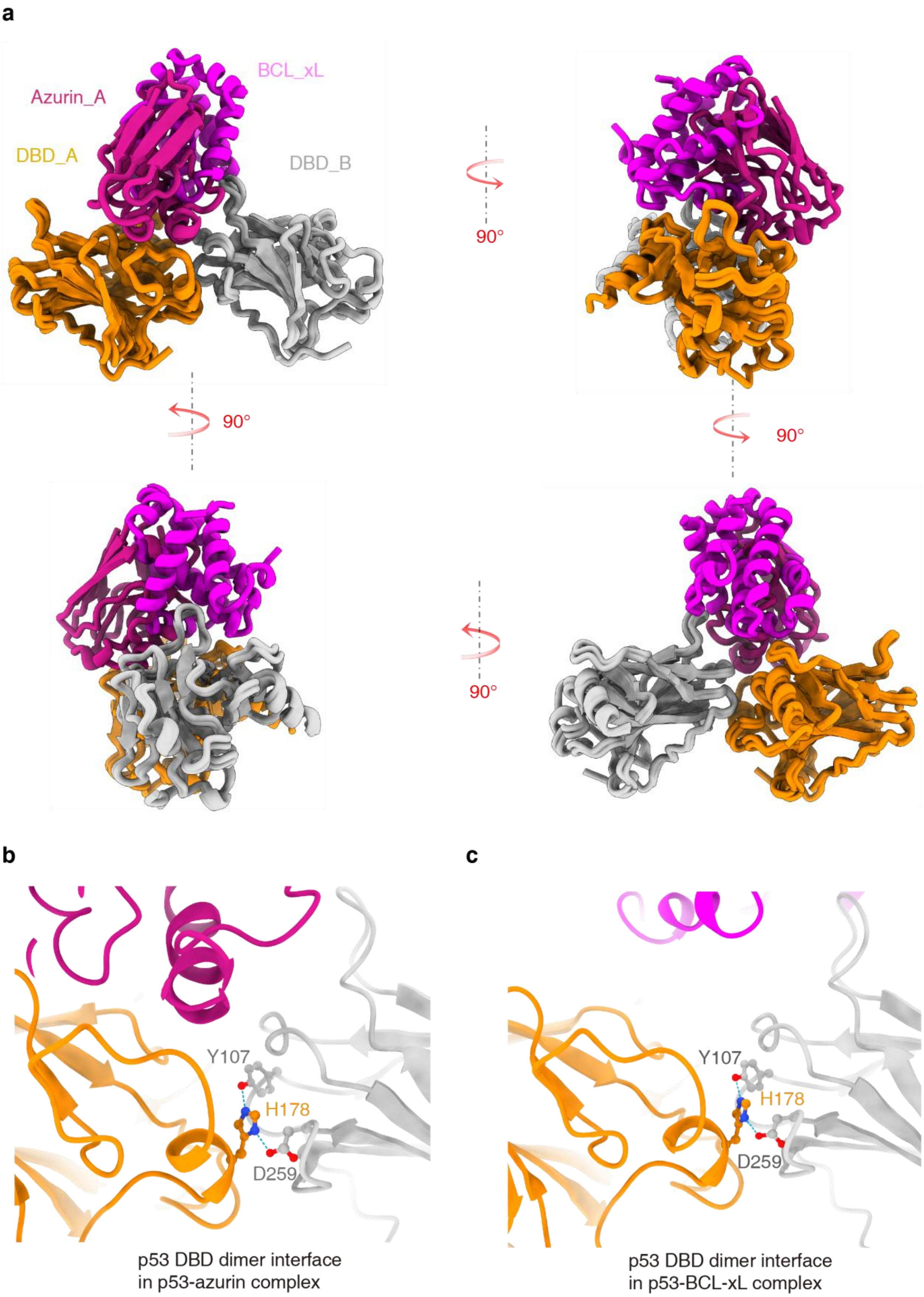
a
BCL_xL
Azurin_A
DBD_A
DBD_B
90°
90°
90°
90°
b
Y107
H178
D259
p53 DBD dimer interface
in p53-azurin complex
c
Y107
H178
D259
p53 DBD dimer interface
in p53-BCL-xL complex

Supplementary Figure 9. Comparison of the structural models of azurin-p53 complex and reported p53-BCL-xL complex.

a Superposition of our azurin-DBD complex with reported BCL-xL in complex with p53-DBD (PDB ID: 6LHD). The two p53-DBD, azurin and BCL_xL are colored in silver, orange, medium violet and light magenta, respectively. **b** Highlighting of the key intermolecular hydrogen bond network at p53-DBD interface of azurin-p53 complex. **c** Key intermolecular hydrogen bond network at p53-DBD interface of p53-BCL-xL complex.

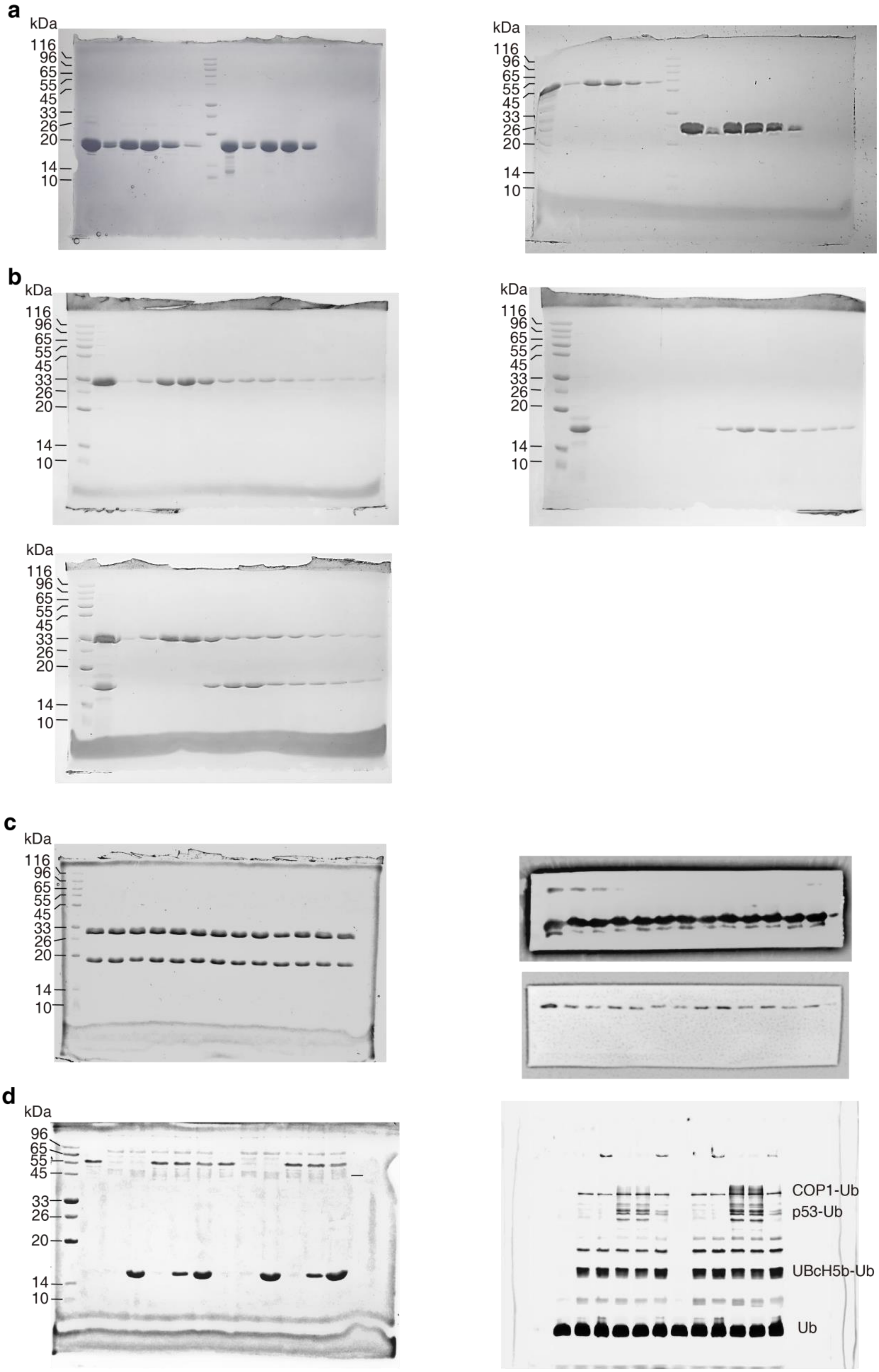
a
kDa
116
96
65
55
45
33
26
20
14
10
b
c
d
COP1-Ub
p53-Ub
UBcH5b-Ub
Ub

Supplementary Figure 10. Original SDS-PAGE gels and Western blots.

a SDS-PAGE gels for supplementary figure 1g, h, i. **b** SDS-PAGE for supplementary figure 1k. **c** SDS-PAGE and western blots for figure 2b. **d** SDS-PAGE and western blots for figure 3c.

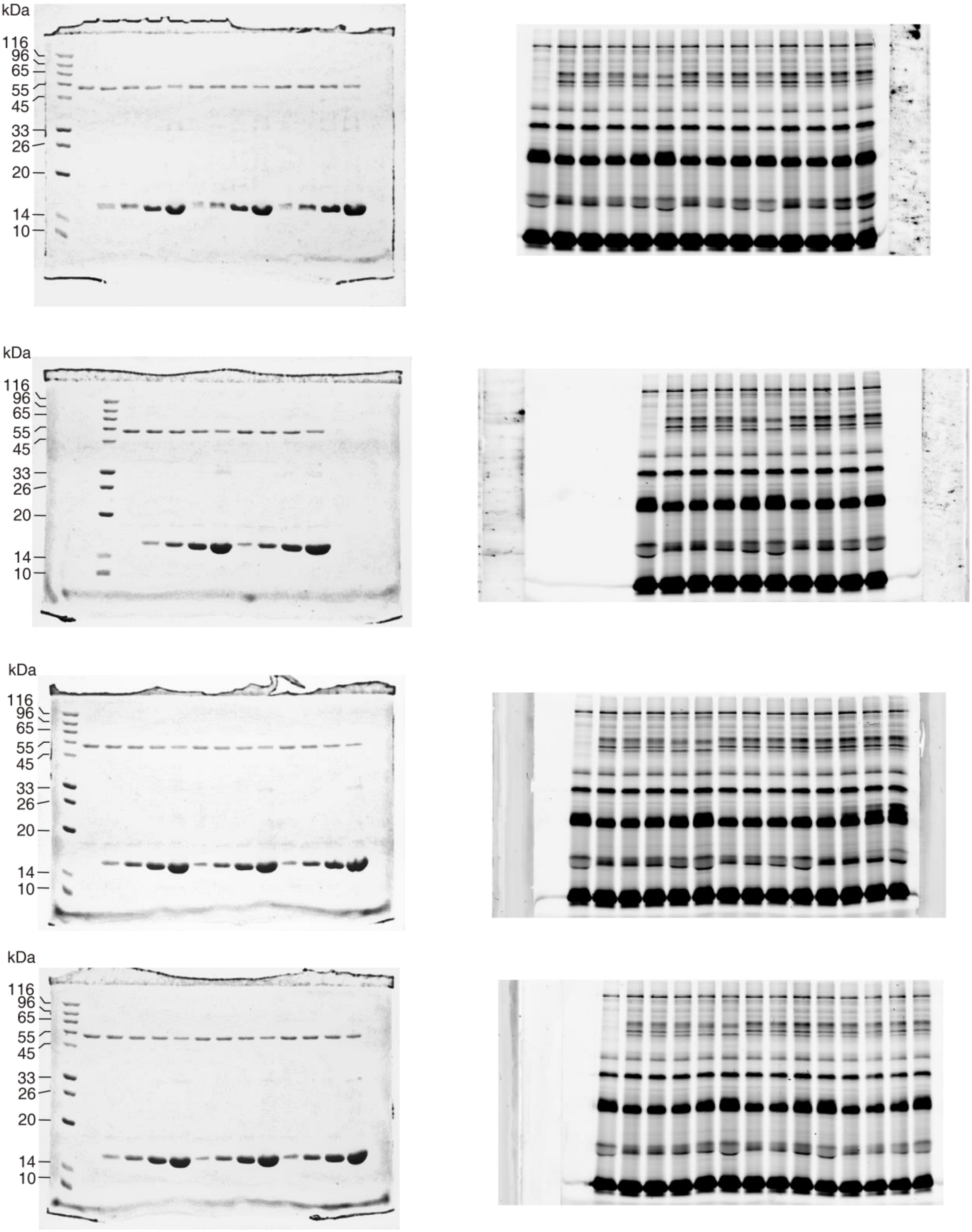

Supplementary Figure 11. Original SDS-PAGE gels and Western blots of ubiquitination assay.

SDS-PAGE gels (left) and western blots with anti-ubiquitin antibody for figure 3d

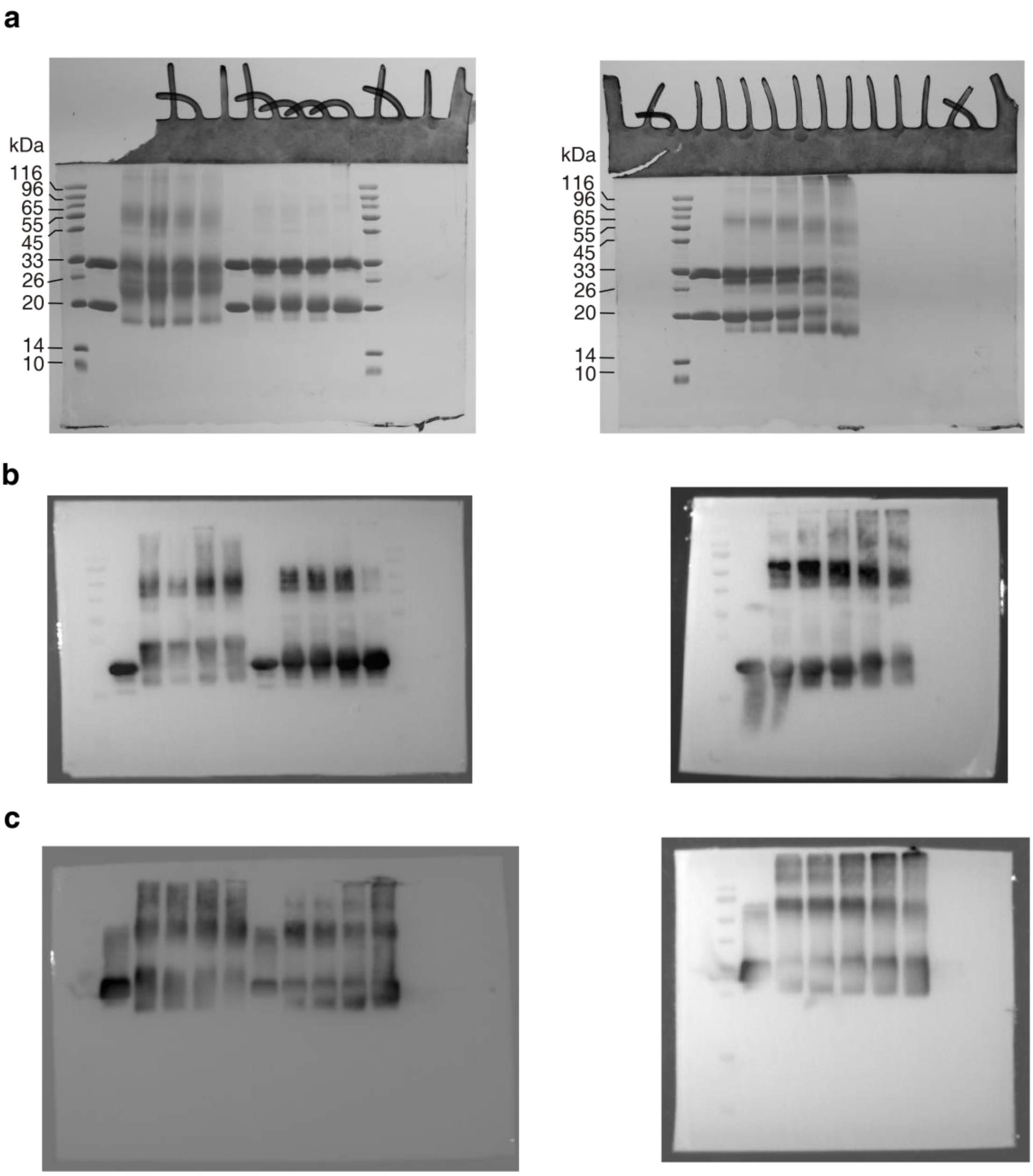

Supplementary Figure 12. Original SDS-PAGE gels and Western blots for crosslinking mass spectrometry analysis.

a SDS-PAGE of p53-DBD and azurin crosslinking with BS3, BS2G and GA presented in supplementary figure 6a. **b** Western blots with anti-strep antibody for supplementary figure 6a. **c** Western blots with anti-flag antibody for supplementary figure 6a.

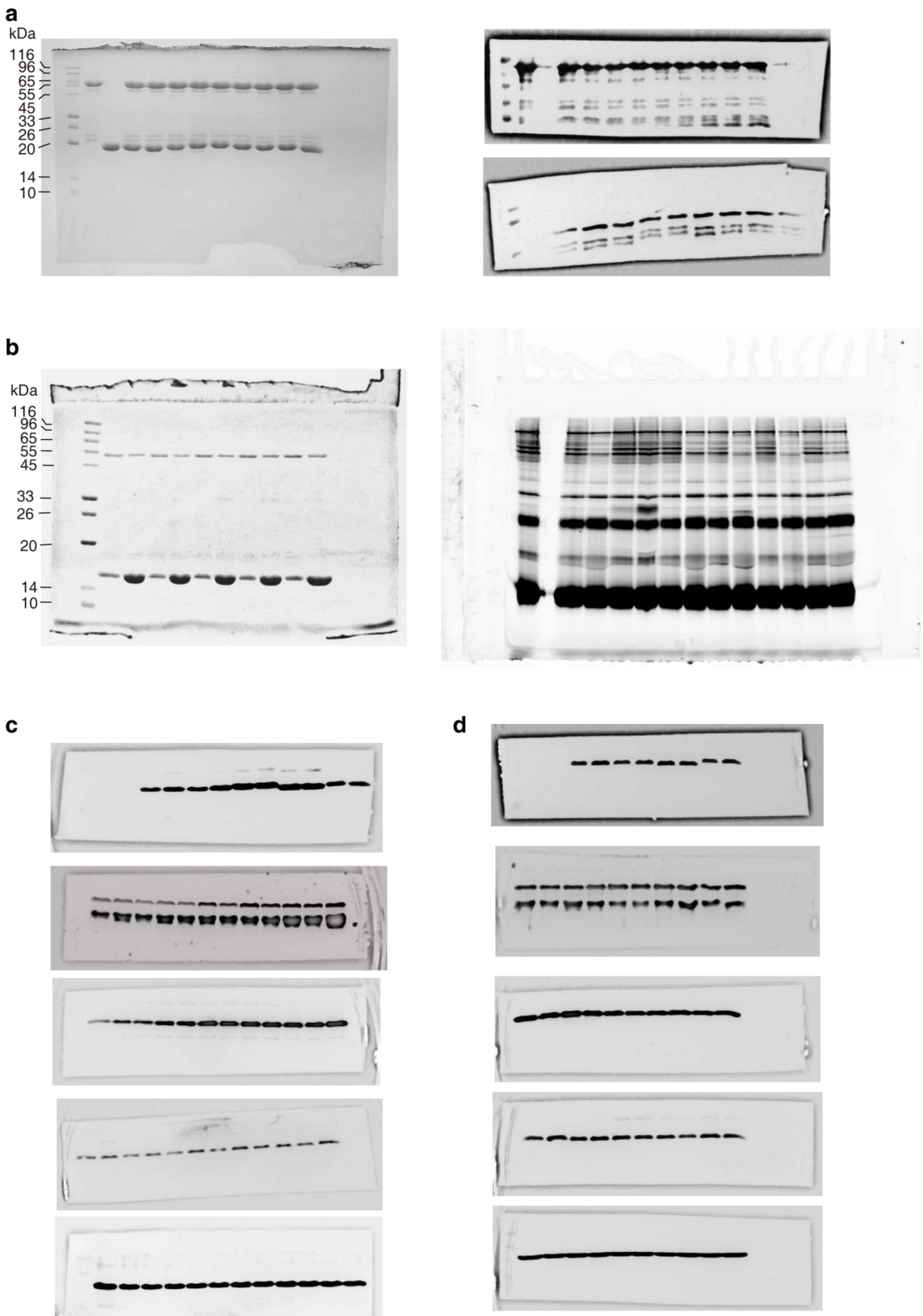
a
kDa
116
96
65
55
45
33
26
20
14
10
b
kDa
116
96
65
55
45
33
26
20
14
10
c
d

Supplementary Figure 13. Original SDS-PAGE gels and Western blots.

a SDS-PAGE and western blots for figure 4b. **b** SDS-PAGE and western blots for figure 4c. **c** Western blots for figure 5c. **d** Western blots for supplementary figure 8b.

Supplementary Table 1. Oligonucleotide primers used in this study.

Primers	Primer sequences (5′ –3′)
**PCR products were cloned in pET15D**	
DBD$_{92-292}$-linker-Azurin-Fw	AAGAAGGAGATATACATATGCCGCTGAGTAGTAGCGTT
DBD$_{92-292}$ linker-Azurin-Rv	TGGTGATGGTGATGCTCGAGTTTTTTGCGCAGATTTTC
linker-Azurin- Fw	CTGGTTCCGCGTGGTAGTGGCGGTGGTGGCAGCGCCGAATGTAGCGTT
linker-Azurin- Rv	ACCACGCGGAACCAGACTACCGCCGCCACCTTTCAGGGTCAGCGT
NTD(1-99)-MBP-Fw	TCCAGGGGCCGCATATGATGGAAGAACCGCAGAGTGAT
NTD(1-99)-MBP-Rv	CAGCCGGATCCTCGAGTTACCAGCTAGGTGCAGG
CTD (293-393)-Fw	AAGTTGATGCACATATGGGTGAACCGCATCATGAG
CTD (293-393)- Rv	CAGCCGGATCCTCGAGTTAATCGCTATCCGGACCTTC
COP1$_{1-731}$-Fw	TCCAGGGGCCGCATATG AGCGGCAGCCGTCAG
COP1$_{1-731}$-Rv	CAGCCGGATCCTCGAGTTA AACCAGTTCCAGAAC
DBD$_{92-292}$-Fw	AAGTTGATGCACATATG GACTACAAAGACCATGACGGTGATTATAAAGATCATGACATC
DBD$_{92-292}$-Rv	CAGCCGGATCCTCGAGTTATTTTTTGCGCAGATTTTC
Azurin$_{1-128}$-Fw	AAGTTGATGCACATATG TGGAGCCACCCGCAGTTC
Azurin$_{1-128}$-Rv	CAGCCGGATCCTCGAGTTA TTTCAGGGTCAGCGTACC
Azurin_ Y72F-Fw	AGCGGTCTGGATAAAGAT TTC CTGAAACCGGATGATAGC
Azurin_ Y72F-Rv	GCTATCATCCGGTTTCAGGAAATCTTTATCCAGACCGCT
Azurin_ Q57R-Fw	AGCACCGCAGCAGATATG CGT GGTGTTGTTACCGATGGT
Azurin_ Q57R-Rv	ACCATCGGTAACAACACCACGCATATCTGCTGCGGTGCT
Azurin_ T61R-Fw	GATATGCAGGGTGTTGTT CGT GATGGTATGGCAAGCGGT
Azurin_ T61R-Rv	ACCGCTTGCCATACCATCACGAACAACACCCTGCATATC
Azurin_ M64E-Fw	GGTGTTGTTACCGATGGT GAA GCAAGCGGTCTGGATAAA
Azurin_ M64E-Rv	TTTATCCAGACCGCTTGCTTCACCATCGGTAACAACACC
Azurin_ G116E-Rv	GGTCAGCGTACCTTTCATCAGTGCGCTATGTTCCGGAAAGGT
Azurin_ M13I-Fw	GCCGAATGTAGCGTTGATATTCAGGGTAATGATCAGATCCAGTTTAACAC C
Azurin_ V43I-Fw	AATCTGCCGAAAAATATCATGGGTCATAATTGG
Azurin_ V43I -Rv	CCAATTATGACCCATGATATTTTTCGGCAGATT
Azurin_ Q57E-Fw	ACCGCAGCAGATATGGAA GGTGTTGTTACCGAT
Azurin_ Q57E -Rv	ATCGGTAACAACACCTTCCATATCTGCTGCGGT
Azurin_ Q57R-Fw	AGCACCGCAGCAGATATG CGT GGTGTTGTTACCGATGGT
Azurin_ Q57R -Rv	ACCATCGGTAACAACACCACGCATATCTGCTGCGGTGCT
Azurin_ S78I-Fw	TATCTGAAACCGGATGATATCCGTGTTATTGCACAT
Azurin_ S78I-Rv	ATGTGCAATAACACGGATATCATCCGGTTTCAGATA
Azurin_ T61D-Fw	GATATGCAGGGTGTTGTT GAT GATGGTATGGCAAGCGGT
Azurin_ T61D-Rv	ACCGCTTGCCATACCATCATCAACAACACCCTGCATATC
Azurin_ G116K-Rv	GGTCAGCGTACCTTTCATCAGTGCGCTATGTTTCGGAAAGGT
Azurin_ A119K-Rv	GGTCAGCGTACCTTTCATCAGTTTGCTATGACCCGGAAA
Azurin_ S100D-Fw	ACCTTTGATGTG GAT AAACTGAAAGAAG
Azurin_ S100D-Rv	CTTCTTTCAGTTTATCCACATCAAAGGT
Azurin_ A119E-Rv	TTTCAGGGTCAGCGTACCTTTCATCAGTTCGCTATG
DBD_ L201G-Fw	ATTCGCGTGGAAGGTAAT GGC CGTGTGGAATATCTGGAT
DBD_ L201G-Rv	ATCCAGATATTCCACACGGCCATTACCTTCCACGCGAAT
DBD_ T150A-Fw	CAGCTGTGGGTTGATAGC GCA CCGCCGCCGGGCACCCGT
DBD_ T150A-Rv	ACGGGTGCCCGGCGGCGGTGCGCTATCAACCCACAGCTG
DBD_ S185A-Fw	CATGAACGTTGTAGTGAT GCA GATGGTCTGGCCCCGCCG
DBD_ S185A-Rv	CGGCGGGGCCAGACCATCTGCATCACTACAACGTTCATG
DBD_ S183M-Fw	CCGCATCATGAACGTTGT ATG GATAGTGATGGTCTGGCC
DBD_ S183M-Rv	GGCCAGACCATCACTATCCATACAACGTTCATGATGCGG
DBD_ S241F-Fw	AATTATATGTGCAACAGC TTT TGCATGGGTGGTATGAAT
DBD_ S241F-Rv	ATTCATACCACCCATGCAAAAGCTGTTGCACATATAATT
DBD_ R248W-Fw	TGCATGGGTGGTATGAAT TGG CGCCCGATTCTGACCATT
DBD_ R248W-Rv	AATGGTCAGAATCGGGCGCCAATTCATACCACCCATGCA
**PCR products were cloned in pET21B**	
p53- Fw	AAGAAGGAGATATACATATGATGGAAGAACCGCAGAGTGAT

p53- Rv	TGGTGATGGTGATGCTCGAGATCGCTATCCGGACCTTC
**PCR products were cloned in pMlink（N-tag）**	
Azurin-N-3xFlag-Fw	GATGACGACGATAAGGGATCCATGGCCGAATGTAGCGTTGAT
Azurin-N-3xFlag-Rv	CTGCTAGCAAGCTTCTCGAGTTATTTCAGGGTCAGCGTACC
Azurin-N-2xStrep-F	CCGCAGTTCGAAAAAGGATCCATGGCCGAATGTAGCGTTGAT
Azurin-N-2xStrep-R	CTGCTAGCAAGCTTCTCGAGTTATTTCAGGGTCAGCGTACC
**PCR products were cloned in pMlink（C-tag）**	
Azurin-C-3xFlag-F	ATTATCGATCCGGAGGTACCATGGCCGAATGTAGCGTTGAT
Azurin-C-3xFlag-R	CATGGTCTTTGTAGTCCTCGAGTTTCAGGGTCAGCGTACC
Azurin-C-2xStrep-F	ATTATCGATCCGGAGGTACCATGGCCGAATGTAGCGTTGAT
Azurin-C-2xStrep-R	GGCTCCAGGCGCTCTCGAGTTTCAGGGTCAGCGTACCTTT
