# Supplementary material for: Structural basis of bacterial effector protein azurin targeting tumor suppressor p53 and inhibiting its ubiquitination
Source: Commun Biol. 2023 Jan 17;6:59. doi: 10.1038/s42003-023-04458-1 (PMC9845241; doi:10.1038/s42003-023-04458-1)
Supplement: Supplementary file 3 — Description of Additional Supplementary Data [file 42003_2023_4458_MOESM3_ESM.docx]

**Description of Additional Supplementary Files**

**File name:** Supplementary Data 1

**Description:** The source data behind the graphs of figure 5b in the paper
