# Supplementary material for: Structural basis of bacterial effector protein azurin targeting tumor suppressor p53 and inhibiting its ubiquitination
Source: Commun Biol. 2023 Jan 17;6:59. doi: 10.1038/s42003-023-04458-1 (PMC9845241; doi:10.1038/s42003-023-04458-1)
Supplement: Supplementary file 5 — Reporting Summary [file 42003_2023_4458_MOESM5_ESM.pdf]

nature portfolio

Corresponding author(s): Qiong Xing

Last updated by author(s): Dec 31, 2022

Reporting Summary

Nature Portfolio wishes to improve the reproducibility of the work that we publish. This form provides structure for consistency and transparency in reporting. For further information on Nature Portfolio policies, see our Editorial Policies and the Editorial Policy Checklist.

Statistics

For all statistical analyses, confirm that the following items are present in the figure legend, table legend, main text, or Methods section.

n/a	Confirmed	
☐	☒	The exact sample size (*n*) for each experimental group/condition, given as a discrete number and unit of measurement
☐	☒	A statement on whether measurements were taken from distinct samples or whether the same sample was measured repeatedly
☐	☒	The statistical test(s) used AND whether they are one- or two-sided<br>*Only common tests should be described solely by name; describe more complex techniques in the Methods section.*
☐	☒	A description of all covariates tested
☐	☒	A description of any assumptions or corrections, such as tests of normality and adjustment for multiple comparisons
☐	☒	A full description of the statistical parameters including central tendency (e.g. means) or other basic estimates (e.g. regression coefficient) AND variation (e.g. standard deviation) or associated estimates of uncertainty (e.g. confidence intervals)
☐	☒	For null hypothesis testing, the test statistic (e.g. *F*, *t*, *r*) with confidence intervals, effect sizes, degrees of freedom and *P* value noted<br>*Give P values as exact values whenever suitable.*
☐	☒	For Bayesian analysis, information on the choice of priors and Markov chain Monte Carlo settings
☐	☒	For hierarchical and complex designs, identification of the appropriate level for tests and full reporting of outcomes
☐	☒	Estimates of effect sizes (e.g. Cohen's *d*, Pearson's *r*), indicating how they were calculated

Our web collection on statistics for biologists contains articles on many of the points above.

Software and code

Policy information about availability of computer code

Data collection	Topspin 3.2, ADDREF, XDS packages,BD FACSuite
Data analysis	HKL2000, NMRPipe, UCSF chimera, ChimeraX, NMRView Java, PyMOL, COOT, Phenix, AMPLE,Amersham typhoon,FlowJo

For manuscripts utilizing custom algorithms or software that are central to the research but not yet described in published literature, software must be made available to editors and reviewers. We strongly encourage code deposition in a community repository (e.g. GitHub). See the Nature Portfolio guidelines for submitting code & software for further information.

Data

Policy information about availability of data

All manuscripts must include a data availability statement. This statement should provide the following information, where applicable:

- Accession codes, unique identifiers, or web links for publicly available datasets
- A description of any restrictions on data availability
- For clinical datasets or third party data, please ensure that the statement adheres to our policy

Atomic coordinate for the p53-azurin structure has been deposited in the Protein Data Bank with accession number 7YGI. Other data if not present in the manuscript or Supplementary information file, are available upon request from the corresponding author.

Human research participants

Policy information about studies involving human research participants and Sex and Gender in Research.

Reporting on sex and gender	This work includes no experiment on live vertebrates or higher invertebrates
Population characteristics	N/A
Recruitment	N/A
Ethics oversight	N/A

Note that full information on the approval of the study protocol must also be provided in the manuscript.

Field-specific reporting

Please select the one below that is the best fit for your research. If you are not sure, read the appropriate sections before making your selection.

☒ Life sciences ☐ Behavioural & social sciences ☐ Ecological, evolutionary & environmental sciences

For a reference copy of the document with all sections, see nature.com/documents/nr-reporting-summary-flat.pdf

Life sciences study design

All studies must disclose on these points even when the disclosure is negative.

Sample size	For the flow cytometry experiment, a total of approximate 106 cells were analyzed for each treatment, and each condition was tested in three independent times. The sample size was determined following the reported work.
Data exclusions	No data excluded
Replication	All attempts at replication were successful.
Randomization	We did not use any sample that need to be randomized
Blinding	All the data collection and analysis were blinded

Reporting for specific materials, systems and methods

We require information from authors about some types of materials, experimental systems and methods used in many studies. Here, indicate whether each material, system or method listed is relevant to your study. If you are not sure if a list item applies to your research, read the appropriate section before selecting a response.

Materials & experimental systems

n/a	Involved in the study
☐	☒ Antibodies
☐	☒ Eukaryotic cell lines
☒	☐ Palaeontology and archaeology
☒	☐ Animals and other organisms
☒	☐ Clinical data
☒	☐ Dual use research of concern

Methods

n/a	Involved in the study
☒	☐ ChIP-seq
☐	☒ Flow cytometry
☒	☐ MRI-based neuroimaging

Antibodies

Antibodies used	anti-DYKDDDDK tag antibody (proteintech, 66008-3-Ig), anti-strep tag antibody (Abbkine, 8C12, ATUMR1201), p21 Waf1/Cip1(Cell signaling ab2974), p53(Proteintech Group 10442-1-AP), Bax (Proteintech Group 60267-1-Ig) ,anti-actin antibody (Proteintech Group)
Validation	There are validation reports for all antibodies we bought and used.

Eukaryotic cell lines

Policy information about cell lines and Sex and Gender in Research

Cell line source(s)	HEK293T Cell line was bought from ABCAM (ab255593).
Authentication	No independent authentication
Mycoplasma contamination	The cell line we used has been tested and confirmed no mycoplasma contamination
Commonly misidentified lines (See ICLAC register)	N/A

Flow Cytometry

Plots

Confirm that:

- [x] The axis labels state the marker and fluorochrome used (e.g. CD4-FITC).
- [x] The axis scales are clearly visible. Include numbers along axes only for bottom left plot of group (a 'group' is an analysis of identical markers).
- [x] All plots are contour plots with outliers or pseudocolor plots.
- [x] A numerical value for number of cells or percentage (with statistics) is provided.

Methodology

Sample preparation	HEK293T Cells (105) were treated with 60 µM azurin and its mutants for 72 h. Cells were washed with PBS and resuspended in binding buffer. Then the cells were treated with a Propidium Iodide (PI)/Fluorescein Isothiocyanate (FITC)-Annexin V Staining Kit (TransGen, FA101) and subjected to flow cytometry analysis.
Instrument	BD FacsVerse
Software	FlowJo software was utilized to calculate the apoptotic cell rate.
Cell population abundance	A total of 106 cells were analyzed for each treatment, and each condition was tested in three independent times.
Gating strategy	We applied the Forward and side scatter gating strategy. The first step is distinguishing populations of cells based on their forward and side scatter properties. Forward and side scatter give an estimation of the size and granularity of the cells respectively,Bivariant analysis of FITC fluorescence and PI fluorescence gave different cell populations, where FITC(-) and PI(-) were designated as viable cells, FITC(+) and PI(-) as apoptotic cells, and FITC(+) and PI(+) as late apoptotic or necrotic cells.

- [ ] Tick this box to confirm that a figure exemplifying the gating strategy is provided in the Supplementary Information.
